# Assessment of Hydration, Nutritional Status and Arterial Stiffness in Hypertensive Chronic Kidney Disease Patients

**DOI:** 10.3390/nu15092045

**Published:** 2023-04-24

**Authors:** Josipa Radić, Ela Kolak, Marijana Vučković, Andrea Gelemanović, Hana Đogaš, Dora Bučan Nenadić, Mislav Radić

**Affiliations:** 1 Internal Medicine Department, Nephrology and Haemodialysis Division, University Hospital Centre Split, 21000 Split, Croatia; mavuckovic@kbsplit.hr (M.V.); hana.dogas@gmail.com (H.Đ.); 2 Department of Internal Medicine, School of Medicine, University of Split, 21000 Split, Croatia; mislavradic@gmail.com; 3 Nutrition and Dietetics Department, University Hospital Centre Split, 21000 Split, Croatia; elakolak93@gmail.com (E.K.); dorabucan@gmail.com (D.B.N.); 4 Mediterranean Institute for Life Sciences (MedILS), 21000 Split, Croatia; andrea.gelemanovic@gmail.com; 5 Internal Medicine Department, Rheumatology, Allergology and Clinical Immunology Division, Center of Excellence for Systemic Sclerosis in Croatia, University Hospital Centre Split, 21000 Split, Croatia

**Keywords:** chronic kidney disease, hydration status, Watson formula, arterial stiffness, 24 h ambulatory blood pressure measurement

## Abstract

The aim of this cross-sectional study was to determine the body fluid volume in patients diagnosed with both chronic kidney disease (CKD) and arterial hypertension (AH), and to investigate the relationship between fluid overload (FO), nutritional status and arterial stiffness in this specific patient population. A total of 169 participants with CKD and AH were enrolled in the study, and data on general parameters, comorbidities, medication use, and laboratory parameters were collected. Body composition was assessed with a Tanita MC 780 device, and data on the central and peripheral systolic and diastolic blood pressure, as well as pulse wave velocity (PWV) and the augmentation index (AIx) were collected with an IEM Mobil-O-Graph 24 h ambulatory blood pressure monitor, which was based on oscillometry. The Mediterranean Diet Serving Score (MDSS) questionnaire was used to determine the adherence to the Mediterranean diet (MeDi). Our results showed that the significant positive predictors of hydration status were the use of diuretics and oral hypoglycemic agents, whereas the negative predictors were female sex, higher body mass index level and use of two or more antihypertensives in the form of a single-pill combination. We also found differences in blood pressure and arterial stiffness parameters in relation to volume status, along with differences based on the presence of diabetes mellitus (DM). In conclusion, these results call for a higher awareness of volume status in the care of CKD patients with AH, especially in those with diabetes mellitus.

## 1. Introduction

Extracellular fluid excess is a common characteristic in patients affected by chronic kidney disease (CKD) causing lower extremity edema, arterial hypertension (AH), pulmonary vascular congestion and heart failure [1,2]. Furthermore, excessive extracellular water is more pronounced in diabetic patients considering the causative relationship between hyperglycemia and hypertonicity [3,4], with the progression of diabetic nephropathy being associated with an increase in extracellular fluid [5]. It has been found that specifically in diabetic CKD patients, the imbalance in the renin–angiotensin–aldosterone system [6] and the increased capillary permeability caused by advanced glycosylation end products (AGEs) [7] increases the risk of fluid overload [3]. Kidney function reduction leads to sodium and water retention, thus resulting in fluid overload (FO) [8] but whether the FO plays a direct role in kidney function decline or is associated with adverse outcomes in CKD is not yet clear [9]. FO is known to increase the circulating volume, venous return and filling pressures, resulting in myocardial systolic and diastolic stress [1]. Furthermore, arterial stiffness evaluated through pulse wave velocity (PWV) accompanies FO and contributes to CKD progression proportionally [9,10]. According to the growing body of evidence, cardiovascular disease (CVA) is the primary cause of morbidity and mortality in CKD [11,12] with FO as one of the main determinants [13,14].

The assessment of FO remains a challenge in clinical practice, considering that the physical signs of edema are of limited value for body hydration status and excess volume determination [11]. For a more accurate assessment of FO as well as a more convenient overall measurement of nutritional status in CKD patients, multifrequency bioelectrical impedance analysis (MF-BIA) has been proposed [15]. MF-BIA measures the difference in the impedance between tissues and body fluid and, as such, can distinguish intracellular and extracellular components [11,16]. The ratio of extracellular water (ECW) and total body water (TBW) in MF-BIA has been commonly used as an indicator of an altered body fluid status but whether this ratio can accurately determine the edematous state is unclear [16]. The mentioned ratio assumes that excess ECW results in edema whereas intracellular water (ICW) remains unaffected by the fluid volume changes. On the other hand, the TBW_BIA_ to TBW_Watson_ ratio may be used for FO assessment considering that the Watson formula provides information about the adequacy of the fluid volume adjusted for age and sex and is derived from the pooled data of healthy subjects [2,16,17].

The Mediterranean diet (MeDi) pattern is associated with a reduction in blood pressure, and the risk of diabetes mellitus (DM), obesity, or CVA disease, which consequently can have a positive effect on CKD with adaptations considering the CKD stage. Among the additional benefits of the adherence to a MeDi in CKD are a reduction in the acid load diet, an improvement in microbiota, a decrease in inflammation and the amelioration of constipation. Furthermore, a higher consumption of plant-based food such as vegetables, fruits, and whole grains, along with a moderate consumption of animal foods contribute to a decrease in the phosphorus and potassium blood levels with a reduction in the consumption of ultra-processed products associated with lower levels of sodium, potassium, and phosphorus [18,19].

The efficacy of the FO assessment using bioimpedance in CKD patients requiring dialysis treatment has been assessed previously but similar data in non-dialytic CKD patients are scarce [20], with only a few studies evaluating the relationship between FO and CVA events or risk factors in this population of patients [11]. The first investigation in this scientific field was the study of Vega et al., who noticed the increased mortality rates in CKD patients with FO [21]. Furthermore, fluid management guided by BIA measurements was found to have a beneficial impact on blood pressure, arterial stiffness and the survival rate in patients undergoing dialysis [11,22,23]. Therefore, the aim of the present research was to determine the body fluid volume in patients diagnosed with both CKD and AH and to investigate the association between FO, arterial stiffness, and nutritional status in this specific population of patients.

## 2. Materials and Methods

### 2.1. Study Population and Design

**Figure 1:**
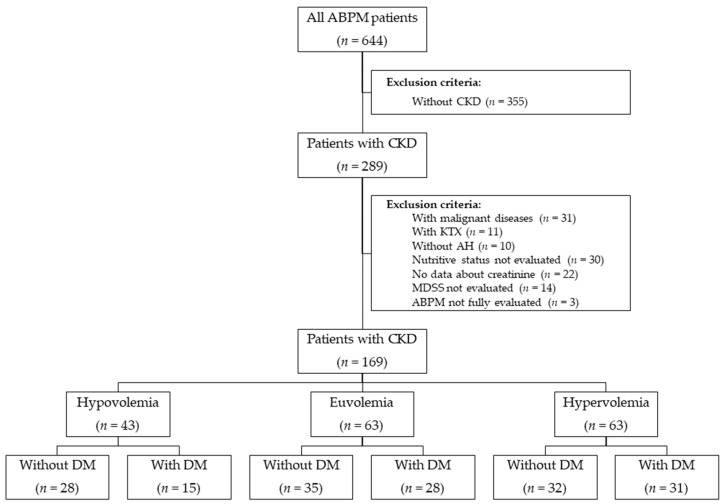
Flowchart of the study. Abbreviations: n—number, CKD—chronic kidney disease, DM—diabetes mellitus, AH—arterial hypertension, KTX—kidney transplant, MDSS—Mediterranean Diet Serving Score, ABPM—ambulatory blood pressure monitoring.

This cross-sectional study was carried out at the Outpatient Clinic for Arterial Hypertension, Nephrology and Haemodialysis Division, Internal Medicine Department, University Hospital Centre Split, Croatia, in the period between February 2021 and December 2022. Out of 644 patients recruited during their regular visit to the nephrologist and dietitian, 289 had CKD defined as estimated glomerular filtration (eGFR) using CKD-EPI ≤ 60 mL/min/1.73 m^2^ and/or albuminuria > 300 mg/24 h. After applying the following exclusion criteria: AH not present; kidney transplantation (KTX); an incomplete or invalid blood pressure and body composition measurement; pregnancy; immobility; implanted pacemaker, cardioverter-defibrillator or stents; limb amputation; existing acute infection or active underlying malignant disease; missing data about eGFR or creatinine; cognitive impairment preventing the completion of questionnaires; refusal to participate in the study, data from 169 participants were eligible for further statistical analysis. The participants were divided based on fluid status, and after that based on the presence of DM. The study flowchart is shown in Figure 1.

All participants were informed of the study’s purpose and gave written and verbal consent.

### 2.2. Body Composition and Fluid Status Measurements

Before body composition measurements, a stadiometer was used to determine the body height of the participants. An MC-780 Multifrequency Segmental Body Mass Analyzer (Tanita, Tokyo, Japan) was used for body composition assessment. All participants were advised beforehand to follow the instructions from the device manual: to empty the bladder, to avoid food or liquid consumption for at least 3 h before the measurement and to refrain from strenuous physical activity and alcohol consumption for at least 1 day before the measurement [24]. The aforementioned scale, which was based on a principle of bioelectrical impedance, was used to assess body mass (kg), body mass index (BMI; kg/m^2^), total body water (TBW; kg), extracellular water (ECW; kg), intracellular water (ICW; kg), muscle mass percentage (%), fat-free mass (kg), fat mass (kg and %), visceral fat, skeletal muscle index (SMI), trunk fat mass (kg and %) and phase angle (°). ECW/TBW ratio was calculated from obtained parameters.

To determine the volume status of participants, TBW_Watson_ according to the Watson formula [18] and TBW_BIA_/TBW_Watson_ ratio were calculated. TBW_BIA_/TBW_Watson_ values <0.970 indicated hypovolemia, values between 0.970 and 1.029 indicated euvolemia and TBW_BIA_/TBW_Watson_ values ≥1.030 indicated hypervolemia.

### 2.3. Blood Pressure and Arterial Stiffness Measurements

To assess blood pressure and arterial stiffness parameters, a 24 h ambulatory blood pressure monitor IEM Mobil-O-Graph (IEM GmbH, Stolberg, Germany), based on the principles of oscillometry, was used. An oscillometric cuff was placed on the non-dominant arm. The device was programmed to obtain blood pressure measurements at intervals of 15 min from 8:01 a.m. to 12:00 p.m. and 30 min from 12:01 p.m. to 8:00 a.m. The recording was determined as valid if it had more than 70% of measurements, precisely a minimum of 20 daytime and 10 nighttime ambulatory blood pressure measurements. The obtained parameters included central systolic (cSBP) and diastolic blood pressure (cDBP), brachial systolic (bSBP) and diastolic blood pressure (bDBP), mean arterial pressure (MAP), heart rate (HR), pulse pressure (PP), stroke volume (SV), cardiac output (CO), total vascular resistance (TVR), reflection magnitude (RM), pulse wave velocity (PWV) and augmentation index (AIx). All participants were advised beforehand to follow the instructions provided by the manufacturer: to keep still until the single measurement was complete with the arm hung loosely or, if sitting, with the lower arm rested on the table or on a support; to place feet flat on the floor when sitting or standing; to refrain from speaking unless to express discomfort and to avoid moving the hand during the measurement [25]. Each study participant underwent a 24 h ambulatory blood pressure measurement (ABPM) on the same day as the body composition measurement and peripheral blood analysis.

### 2.4. Mediterranean Diet Serving Score (MDSS)

A semiquantitative food frequency questionnaire, MDSS, was administered to assess the dietary habits and adherence to the Mediterranean diet (MeDi) principles in each participant. This validated and easily applicable questionnaire was based on the frequency of consumption of 14 different food items as well as food groups [26]. Three points were allocated for consumption of MDSS components such as cereals, olive oil, vegetables and fruit with every meal, two points were allocated for daily consumption of dairy products and nuts; whereas one point was allocated based on weekly consumption of potatoes (≤3), legumes (≥2), eggs (2–4), poultry (2), red meat (<2), fish (≥2), sweets (≤2) and fermented beverages (1 and 2 glasses a day for females and males, respectively) [27]. For intakes higher or lower than recommended for any of the 14 MDSS components, 0 was allocated. Therefore, MDSS ranged from 0 to 24 with values higher than 13.5 indicating adherence to the MeDi principles.

### 2.5. Medical History, Clinical and Laboratory Parameters

For each study participant, data about the duration of CKD, AH and DM, the presence of other comorbid conditions and data related to the use of medication were collected.

Regarding laboratory parameters, blood samples in fasting conditions were taken before 24 h ABPM monitor placement for each study participant. Obtained data included erythrocyte count (×10^12^/L), hemoglobin (Hb; g/L), mean corpuscular volume (MCV; fL), urea (mmol/L), creatinine (µmol/L), estimated glomerular filtration rate (eGFR; mL/min/1.73 m^2^), fasting blood glucose (FBG; mmol/L), hemoglobin A1c (HbA1c; %), uric acid (µmol/L), sodium (mmol/L), potassium (mmol/L), neutrophil count (%), lymphocyte count (%), and leukocyte count (×10^9^/L).

### 2.6. Statistical Analysis

First, a Shapiro–Wilk test was performed to assess the normality of the data. If the numerical data were following a normal distribution, they were presented with mean and standard deviation (SD); otherwise, median, and interquartile range (IQR) were used. Categorical data were presented as numbers and percentages. To examine the difference among groups, a chi-square test was used for categorical data, whereas one-way ANOVA or t-test, and Kruskal–Wallis or Mann–Whitney U test were used for numerical data depending on the normality distribution and the number of groups being compared. When comparing three groups based on their hydration status, adequate post hoc tests were implemented (post hoc chi-square test, Tukey’s HSD or Dunn’s test) depending on the data type and normality distribution. To assess the correlation between hydration status, nutritional parameters, MeDi, blood pressure and arterial stiffness parameters, Spearman’s rank correlation was calculated. Finally, to identify the predictors associated with hydration status, first, a LASSO regression analysis was performed as a feature selection algorithm, which was a penalized regression and accounted for situations with numerous colinear variables. The selected variables were then used as independent variables in multivariate linear regression. Results of linear regression were presented with a beta coefficient (β) and standard error (SE). Statistically significant results were those with a *p*-value < 0.05. The entire statistical analysis was performed using the free software environment for statistical computing R version 4.0.0 [28].

## 3. Results

**Figure 2:**
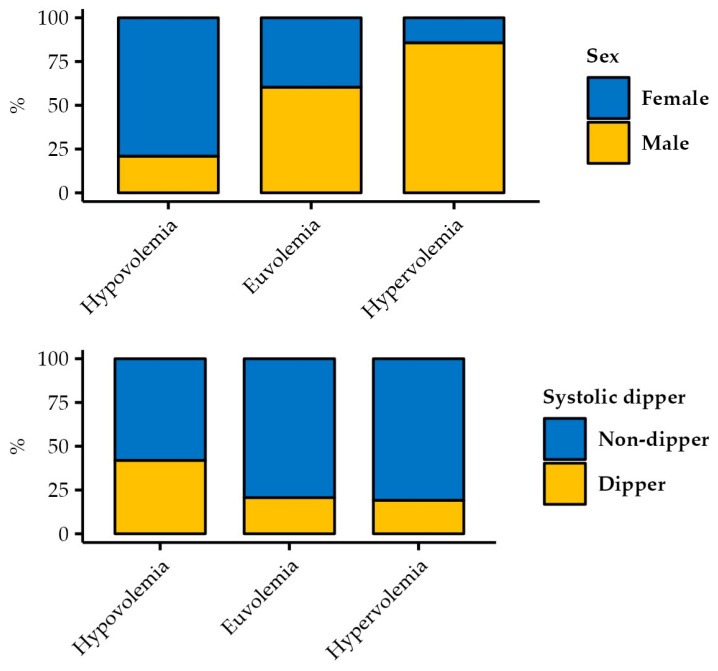
Significant differences in sex and systolic dipping status regarding fluid status.

**Figure 3:**
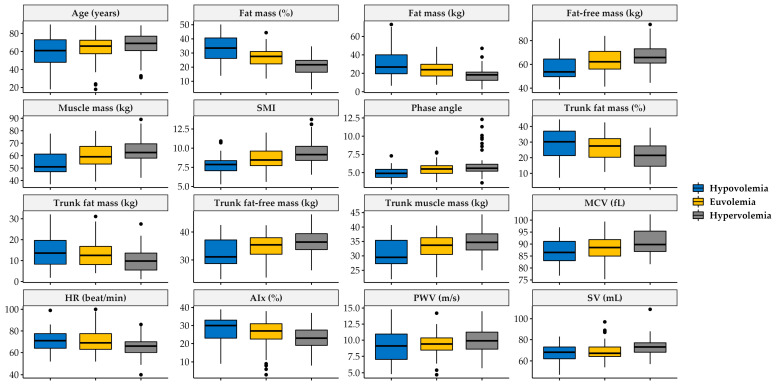
Significant differences in basic characteristics and measured clinical parameters based on the hydration status. Abbreviations: SMI—skeletal muscle index, MCV—mean corpuscular volume, HR—heart rate, AIx—augmentation index, PWV—pulse wave velocity, SV—stroke volume.

After applying extensive exclusion criteria, 169 participants diagnosed with CKD and AH were included in the present study, with a median age of 66 years (IQR = 16), of whom 40% were women. According to the TBW_BIA_/TBW_Watson_ ratio, hypovolemia was determined in 25.4% (43), whereas euvolemia and hypervolemia were equally determined in 37.3% (63) of the included participants. The descriptive statistics for all measured clinical parameters are shown in Table S1 with the participants were grouped into three categories based on their hydration status following the TBW_BIA_/TBW_Watson_ ratio (hypovolemia, euvolemia or hypervolemia). Overall, the participants with hypervolemia were predominantly male, significantly older, had more fat-free mass, muscle mass, SMI, and a higher phase angle. Regarding the BMI, all participants were overweight with the lowest values observed in the hypervolemic participants, but the observed difference did not reach a significance level. Regarding the biochemical parameters, only Hb, MCV and uric acid were shown to be elevated in the participants with hypervolemia. Regarding the blood pressure and arterial stiffness parameters, the participants with hypervolemia had significantly higher PWV and SV values, but lower HR and AIx values. Furthermore, the hypovolemic participants had significantly higher systolic blood pressure dipping rates. All of the significant results regarding the volume status are also presented graphically in Figure 2 and Figure 3.

Out of the 169 participants, only 14 (8.3%) showed adherence to the principles of the MeDi according to the MDSS questionnaire with an overall median MDSS score of 7 (IQR = 5). A significantly higher adherence to the recommended alcohol consumption was noticed in the participants with hypervolemia as shown in Table S2. None of the other MDSS individual items were shown to be statistically different among the participants.

Considering the impact that hyperglycemia has on cellular water distribution, the participants were further divided into two groups based on the presence of DM. Out of the 169 total participants, 74 (43.8%) were affected by DM. The differences among all of the measured clinical parameters were evaluated separately for each category of the hydration status to assess the influence of DM. The statistically significant results are shown in Table 1, whereas the full results are shown in Table S3. The only one parameter that was identified as statistically significant, regardless of hydration status, was a higher FBG in the participants with DM. In the euvolemic group, those with DM had significantly higher visceral fat, and lower values of bDBP, MAP, cSBP and cDBP. In the hypovolemic group, those with DM had significantly higher BMI and SMI levels and were slightly more adherent to the recommended intake of sweets. Interestingly, in the group of hypovolemic participants, none of the participants adhered to the principles of the MeDi no matter the presence of DM. Finally, in the hypervolemic group, the participants with DM had significantly higher values of PP and were also slightly more adherent to the recommended intake of sweets.

Additionally, we evaluated the effect of the CKD stage and eGFR levels based on the hydration status and the results are depicted in Supplementary Figures S1–S6. Interestingly, neither the CKD stage nor the level of eGFR were shown to be significantly associated with the hydration status evaluated as the TBW_BIA_/TBW_Watson_ ratio. Due to this, we compared all other measured and calculated parameters concerning the hydration status and found that the ICW and TBW_Watson_ levels were significantly lower with a higher CKD stage; however, these findings were observed only in the subgroup of participants suffering from DM. When looking at the correlations with the eGFR levels, the above-mentioned finding was replicated only for the TBW_Watson_ levels (positive correlation with eGFR levels).

**Figure 4:**
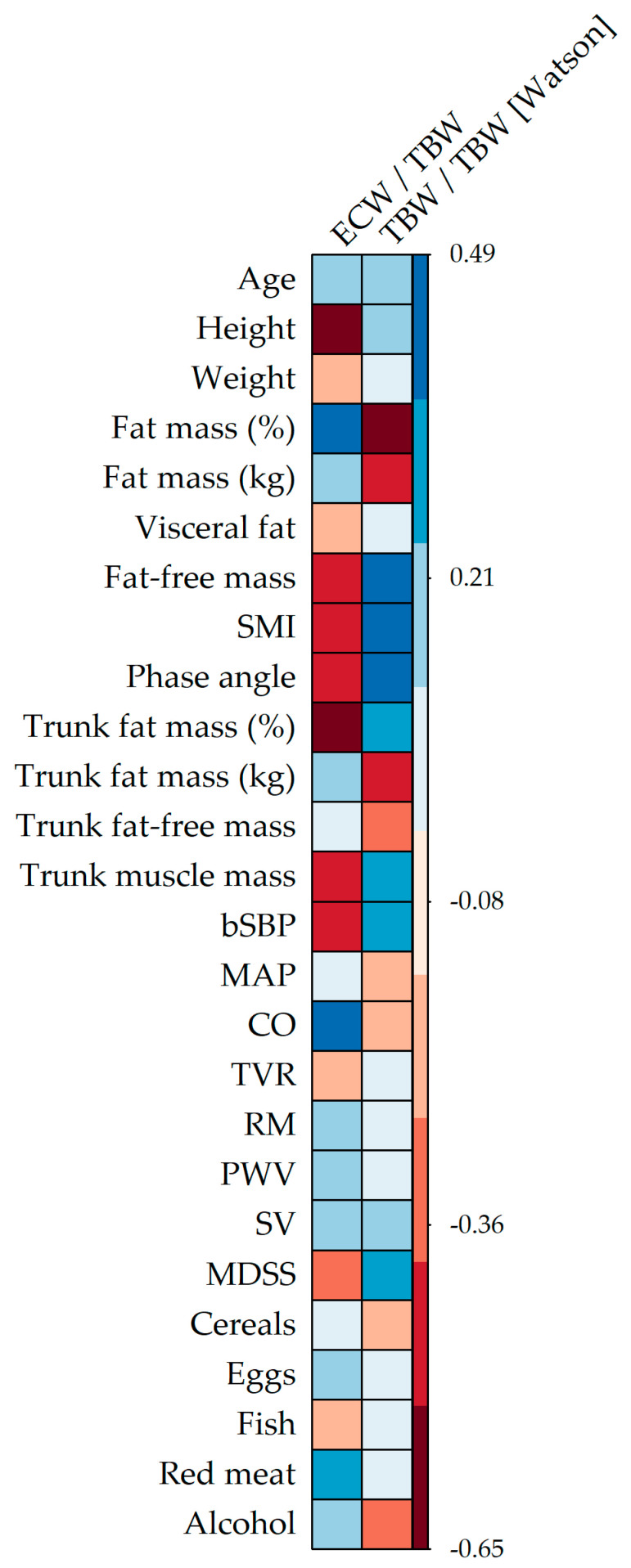
Correlations between all hydration status parameters with regards to other nutritional, blood pressure, arterial stiffness and MeDi parameters. Abbreviations: SMI—skeletal muscle index, bSBP—brachial systolic blood pressure, MAP—mean arterial pressure, CO–cardiac output, TVR—total vascular resistance, RM–reflection magnitude, PWV–pulse wave velocity, SV–stroke volume, MDSS–Mediterranean Diet Serving Score.

The correlations between all of the hydration status parameters with regards to the other nutritional, blood pressure, arterial stiffness and MeDi parameters are shown in Figure 4. Overall, positive correlations were found for age, fat-free mass, SMI, phase angle, bSBP, SV and MDSS, whereas negative correlations were found for fat mass, MAP, CO, cereals intake adherence and wine intake adherence according to the MDSS questionnaire.

As shown in Table 2, to identify the most relevant predictors of hydration status according to the TBW_BIA_/TBW_Watson_ ratio while considering all of the measured clinical parameters, a regression analysis was performed in two steps. First, a penalized LASSO regression analysis was performed, which selected the most relevant predictors and solved the issue of the large collinearity between the parameters, and then a multivariate linear regression was performed to assess the significance of each retained parameter in the model. The significant positive predictors of hydration status in accordance with the TBW_BIA_/TBW_Watson_ ratio were the intake of diuretics and oral hypoglycemics, whereas the negative predictors were female gender, higher levels of BMI and intake of two or more antihypertensives as a single-pill combination. In addition, the final model also retained the following parameters without reaching statistical significance: age, SV, intake of beta-blockers (BB), angiotensin receptor blocker (ARB), moxonidine and vegetable consumption adherence, which were positive predictors according to the MDSS questionnaire; while PWV, systolic dipper, intake of angiotensin-converting enzyme (ACE) inhibitors and egg consumption adherence were negative predictors according to the MDSS questionnaire. Overall, this model explained 23.7% of the variance.

## 4. Discussion

The present study evaluated the hydration status of participants affected by both DM and AH using noninvasive BIA and its association with arterial stiffness, dietary habits, and nutritional status. A total 63 (37.3%) participants met the criteria for hypervolemia according to the TBW_BIA_/TBW_Watson_ ratio, which was less than reported in other studies [16,22,29,30,31]. These differences arose from the specificity of the populations included (i.e., age, sex, body composition and comorbidities) and the way of estimating overhydration (i.e., ECW/TBW ratio, OH/ECW ratio). In terms of the general parameters and the differences according to the hydration status, more women were hypovolemic, and the participants with an older age were more likely to be hypervolemic, which was to be expected because of declining renal and cardiac function with age. The explanation for the susceptibility of the female sex to hypovolemia might be due to the physiologically higher fat mass fraction in females. The differences in body composition with respect to volume status were largely expected and self-explanatory. An interesting difference was found in the phase angle, which contradicted the results of other studies [31]. The difference in the results might arise from the specificity of the population included in this research. In the current study, predominantly men were observed in the hypervolemia group, and therefore, a higher proportion of muscle mass was observed, which was associated with a higher phase angle value [32,33].

Regarding the ABPM parameters, our results suggested a higher heart rate in the hypovolemic participants, which could be explained by the sympathetic response to hypovolemia. The higher estimated stroke volume in the hypervolemic participants was probably due to the higher end-diastolic volume because of the overall hypervolemia. In addition, we found higher values of PWV in the hypervolemic participants, although it should be noted that there were age differences between the groups that could explain the differences. Regarding arterial stiffness, significant differences were found in the heart rate-corrected AIx 75, with the euvolemic participants having higher values than the hypervolemic participants. It is known from the literature that AIx is considered a more sensitive marker of arterial stiffness in younger individuals, whereas PWV is a better measure in older individuals, and these differences might also be due to the age effect in our population [34]. The hypovolemic CKD participants were also more likely to be dippers for the SBP.

Regarding the laboratory parameters, higher Hb and MCV values were found in the hypervolemic CKD participants with AH. This could be due to the different anemia treatment in each group of participants, which we unfortunately did not consider. We also did not consider vitamin B12 and folic acid levels when performing this study.

When discussing the differences in our population in terms of the presence of DM, we found higher levels of BMI, visceral fat, SMI, and trunk fat-free mass, along with a higher metabolic age; all of which were indicative of obesity in the participants with DM in our population compared with nondiabetics. The results of recent longitudinal studies indicated that a metabolically unhealthy body composition characterized by a high proportion of fat mass was not only a risk factor for the development of DM but could also be a consequence of the disease itself [35], further highlighting the importance of adequate and timely lifestyle changes. Higher FBG and Hba1c levels were also higher in diabetics, as expected. The leukocyte counts were also higher in diabetics, which might be due to the higher proportion of adipose tissue known to cause low-grade inflammation [36]. The higher presence of CVA disease in diabetics found in our results was consistent with previous findings and suggested a high CV risk in diabetics [37].

Regarding the parameters of blood pressure and arterial stiffness, our results suggested lower levels of bDBP, MAP, cSBP, cDBP and TVR in the diabetic patients. Hypertension management in diabetic patients is specific and challenging for clinicians. Glycemic control, SBP and DBP levels, together with ageing and neuronal death play an important role in the development of diabetes-associated cardiac autonomic neuropathy (CAN) [38]. CAN is responsible for blood pressure variability and orthostatic hypotension in diabetics. CAN often manifests first in the vagus nerve, resulting in decreased parasympathetic tone [39]. This could be the explanation for our mentioned findings in the participants with DM.

An exceptionally low adherence to the MeDi was observed among the study participants regardless of their volume status and the presence of DM. The MeDi, characterized primarily by a high intake of fruits, vegetables, breads and whole grains, potatoes, beans, nuts, and seeds, is known to be beneficial for reducing CV risk, risk of developing DM, obesity and cognitive health in the general population. A knowledge of the benefits of the MeDi in the CKD participant group is growing, but considering the specificity of the primary disease, precautions are especially important in the selection of low-potassium fruit and vegetable alternatives and in preparation to reduce potassium [20]. As expected, the participants diagnosed with DM had better adherence to the recommended sweets intake according to the MeDi when compared to the participants without DM, considering that sweets consumption was usually associated with hyperglycemia. The obtained result was mostly related to the perception and overall knowledge of patients about foods that could cause an increase in blood glucose [40].

The prescription of diuretics and oral hypoglycemic agents proved to be a positive predictor of hydration status in our study. Diuretics are commonly prescribed and, unfortunately, we did not consider the dose of the diuretics or the type of diuretic. We could only hypothesize that the participants with heart failure and CKD were more frequently prescribed diuretics because of their volume overload. Part of the explanation could be treatment adherence, which we, unfortunately, did not consider in the conduct of this study. A higher intake of oral hypoglycemics as a positive predictor of hydration status could reflect poorly regulated glycemia [41,42]. Among the negative predictors of volume status were female gender and BMI. High BMI values are most often a reflection of an unfavorable body composition, i.e., an increased proportion of fat mass. Furthermore, the differences in body composition regarding gender, where the fat mass is represented in a higher percentage in women, are well known, which is why the results were obtained in accordance with the expectations [43,44]. Being prescribed two or more antihypertensives in a single-pill combination was found to be a negative predictor of hydration status. Common combinations of two or more antihypertensives include ACE inhibitors or ARBs and diuretics. ACE inhibitors with known natriuretic and diuretic effects might be responsible for these results. Furthermore, adherence to the prescribed medication decreases with the number of pills a patient needs to take. A combination of substances in one single pill may increase the adherence and lead to a better clinical outcome, in this case better regulation of blood pressure and volume status. [45,46].

The limitations of our study arose primarily from the cross-sectional design, which prevented us from drawing causal conclusions. One of the limitations was also that salt intake was not assessed when conducting this study, which could affect the fluid status and management. In addition, the dose and type of diuretic were not considered when conducting this study. Another limitation of this study was that the management of anemia in this particular patient population and vitamin B12 and folic acid levels were not considered in the conduct of this study.

## 5. Conclusions

As this study results showed, the correlations between hydration status and nutritional, blood pressure, arterial stiffness and MeDi parameters were found. Furthermore, significant positive predictors of hydration status were the intake of diuretics and oral hypoglycemics, while female gender, higher levels of BMI and intake of two or more antihypertensives as a single-pill combination were negative predictors. Overall, considering all stated, there should be a better awareness of the volume status in the care of CKD patients with AH. To better monitor CKD patients, measurements of body composition with a special focus on fluid status are advised and could be included in standard care for this patients’ population. Further prospective studies on fluid management are needed for a better understanding and treatment of CKD patients with AH.

## Figures and Tables

**Table 1:** Statistically significant parameters regarding the presence of diabetes mellitus and hydration status of participants diagnosed with CKD and AH.

Category	Hypovolemia			Euvolemia			Hypervolemia		
	Without DM (*n* = 28)	With DM (*n* = 15)	p *	Without DM (*n* = 35)	With DM (*n* = 28)	p *	Without DM (*n* = 32)	With DM (*n* = 31)	p *
BMI (kg/m^2^), median (IQR)	27.77 (6.46)	33.47 (9.01)	0.021	27.2 (7.05)	28.6 (3.85)	0.083	27.2 (4.38)	28.44 (4.2)	0.256
Visceral fat level, median (IQR)	7.5 (4.25)	10 (9)	0.050	9.31 (4.48)	12 (5.57)	0.038	9.09 (4.08)	10.61 (4.07)	0.144
Metabolic age (years), median (IQR)	52.21 (17.19)	63.87 (12.02)	0.025	52.94 (15.5)	62.82 (15.69)	0.015	53.12 (13.77)	58.06 (12.26)	0.138
SMI, median (IQR)	7.48 (1.01)	8.49 (1.49)	0.012	8.41 (1.24)	8.98 (1.4)	0.089	9.16 (1.76)	9.16 (2.09)	0.532
Trunk fat-free mass (kg), mean (SD)	31.28 (4.96)	34.43 (4.56)	0.048	35 (5.95)	35.85 (5.12)	0.515	36.65 (4.02)	36.46 (4.04)	0.851
FBG (mmol/L), median (IQR)	5.25 (1.12)	8.95 (6.85)	0.001	5.6 (1.47)	7.95 (1.93)	0.002	6.25 (1.05)	7.3 (1.7)	0.017
HbA1c (%), median (IQR)	5.1 (NA)	8 (2.23)	0.301	6.05 (0.64)	7.07 (1.07)	0.212	5.79 (0.91)	7.05 (0.95)	0.014
Leukocyte count (×10^9^/L), median (IQR)	8 (3.55)	11.05 (8.15)	0.223	7.45 (2.55)	8 (1.85)	0.098	6.8 (1.7)	8.4 (3.4)	0.016
Presence of CVA disease, #*n* (%)	1 (3.85)	6 (40)	0.011	6 (17.14)	13 (46.43)	0.025	10 (31.25)	9 (29.03)	1.000
bDBP (mmHg), mean (SD)	78 (11.44)	78.2 (6.04)	0.950	84.31 (10.61)	74.18 (9.26)	<0.001	77.75 (13.57)	78.71 (9.02)	0.743
MAP (mmHg), median (IQR)	102.5 (13.9)	105.07 (11.27)	0.543	106.74 (10.2)	98.54 (10.97)	0.003	100.94 (13.8)	106 (14.15)	0.156
PP (mmHg), median (IQR)	52 (14.25)	55 (11.5)	0.339	47 (9.5)	51 (12.75)	0.098	50 (9.75)	54 (18.5)	0.018
cSBP (mmHg), median (IQR)	116 (20)	121 (10)	0.251	122.34 (10.69)	115.82 (14.11)	0.041	117.5 (18.75)	121 (18)	0.259
cDBP (mmHg), mean (SD)	80.04 (12.14)	80.27 (6.16)	0.945	86.23 (10.61)	75.57 (9.6)	<0.001	79.34 (13.78)	80.35 (9.76)	0.739
TVR (s/cm^2^), median (IQR)	1753 (274.25)	1733 (148)	0.889	1807.97 (158.36)	1713.21 (192.58)	0.036	1766.5 (216.75)	1707 (271)	0.978
Non-adherence to the MeDi, *n* (%)	28 (100)	15 (100)	0.047	32 (91.43)	24 (85.71)	0.754	28 (87.5)	28 (90.32)	1.000
Adherence to sweets recommendation, *n* (%)	12 (42.86)	12 (80)	0.044	17 (48.57)	19 (67.86)	0.200	18 (56.25)	26 (83.87)	0.035

* *p*-values were obtained with a chi-square test for categorical data, one-way ANOVA for parametric numerical data and Kruskal–Wallis for non-parametric numerical data. Abbreviations: BMI—body mass index, SMI—skeletal muscle index, FBG—fasting blood glucose, HbA1c—hemoglobin A1c, CVA – cardiovascular disease, CKD—chronic kidney disease, bDBP—brachial diastolic blood pressure, MAP—mean arterial pressure, PP—pulse pressure, cSBP—central systolic blood pressure, cDBP—central diastolic blood pressure, TVR—total vascular resistance, MeDi—Mediterranean diet, NA- non applicable.

**Table 2:** Predictors of hydration status according to TBW_BIA_/TBW_Watson_ ratio in participants diagnosed with CKD and AH.

Predictor	Beta	SE	*p*
Sex (female) *	−0.049	0.012	**<0.001**
Age (years)	0.001	0.001	0.561
BMI (kg/m^2^) *	−0.002	0.001	**0.024**
PWV (m/s)	−0.001	0.008	0.866
SV (mL)	0.000	0.001	0.589
Systolic dipper	−0.017	0.013	0.189
BB use	0.023	0.012	0.051
ACE inhibitors use	−0.018	0.012	0.128
ARB use	0.007	0.017	0.686
Moxonidine use	0.015	0.012	0.208
Diuretics use *	0.026	0.012	**0.028**
**Oral hypoglycemic medication use** *	0.024	0.012	**0.040**
≥2 antihypertensives as a single-pill combination *	−0.037	0.014	**0.009**
Adherence to recommended vegetable consumption	0.009	0.014	0.526
Adherence to recommended eggs consumption	−0.018	0.011	0.098

* Bolded are statistically significant predictors. Abbreviations: BMI—body mass index, PWV—pulse wave velocity, SV—stroke volume, BB—beta blockers, ACE—angiotensin-converting enzyme, ARB—angiotensin receptor blocker.

## Data Availability

Raw data can be found at corresponding author via e-mail: josiparadic1973@gmail.com.

## Supplementary Materials

The following supporting information can be downloaded at: https://www.mdpi.com/article/10.3390/nu15092045/s1, Supplementary Table S1. Basic characteristics and measured clinical parameters based on the hydration status of participants diagnosed with CKD and AH, Supplementary Table S2 Adherence to the Mediterranean diet according to the hydration status, Supplementary Table S3. Differences in hydration status according to the presence of diabetes mellitus, Figures S1–S6; CKD stage and eGFR levels regarding hydration status.
