# Supplementary figures and images for: Assessment of Hydration, Nutritional Status and Arterial Stiffness in Hypertensive Chronic Kidney Disease Patients
Source: Nutrients. 2023 Apr 24;15(9):2045. doi: 10.3390/nu15092045 (PMC10180629; doi:10.3390/nu15092045)

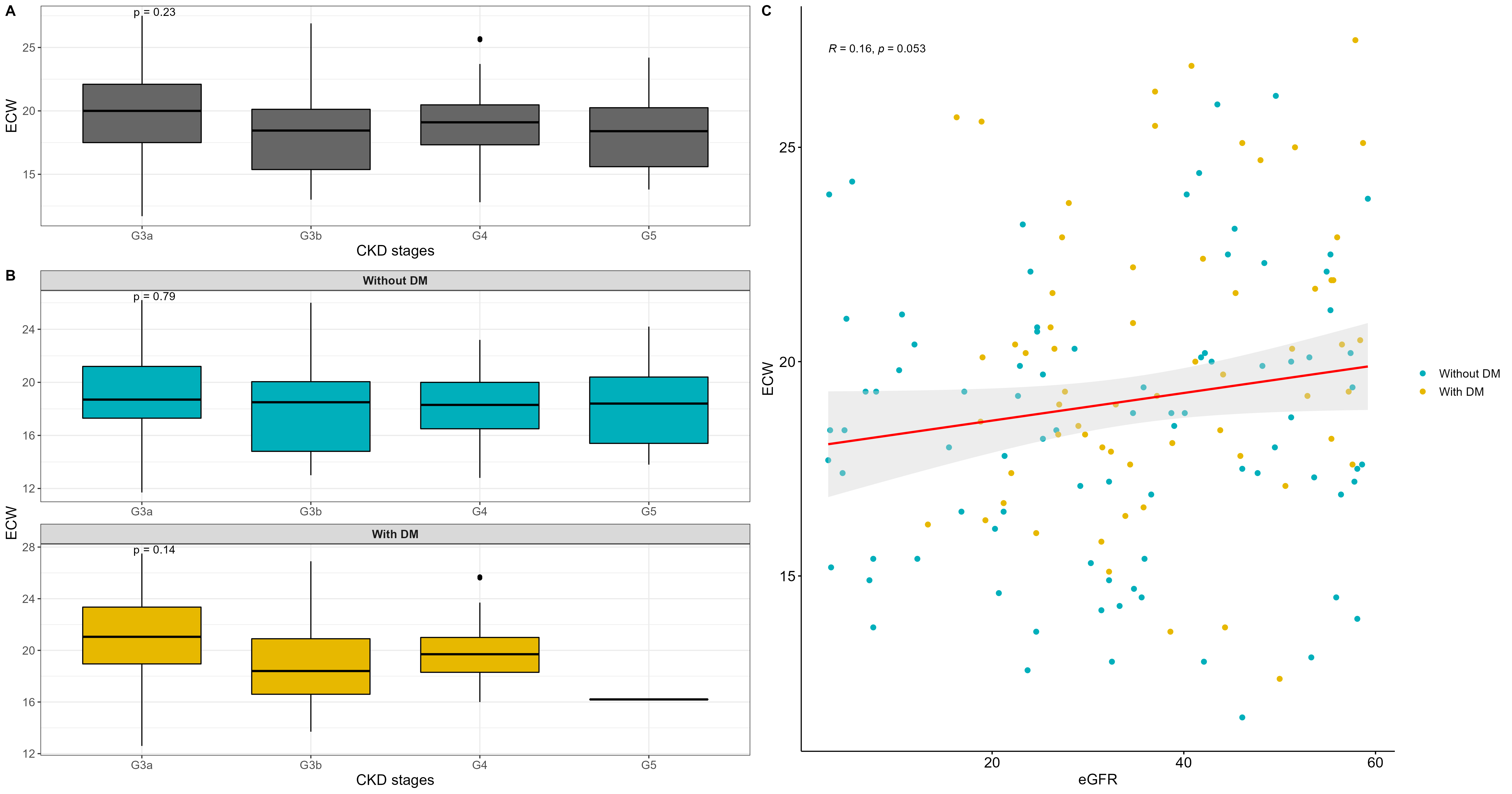

Supplement: Supplementary file 1 [file nutrients-15-02045-s001.zip › CKDstats_ECW.png]

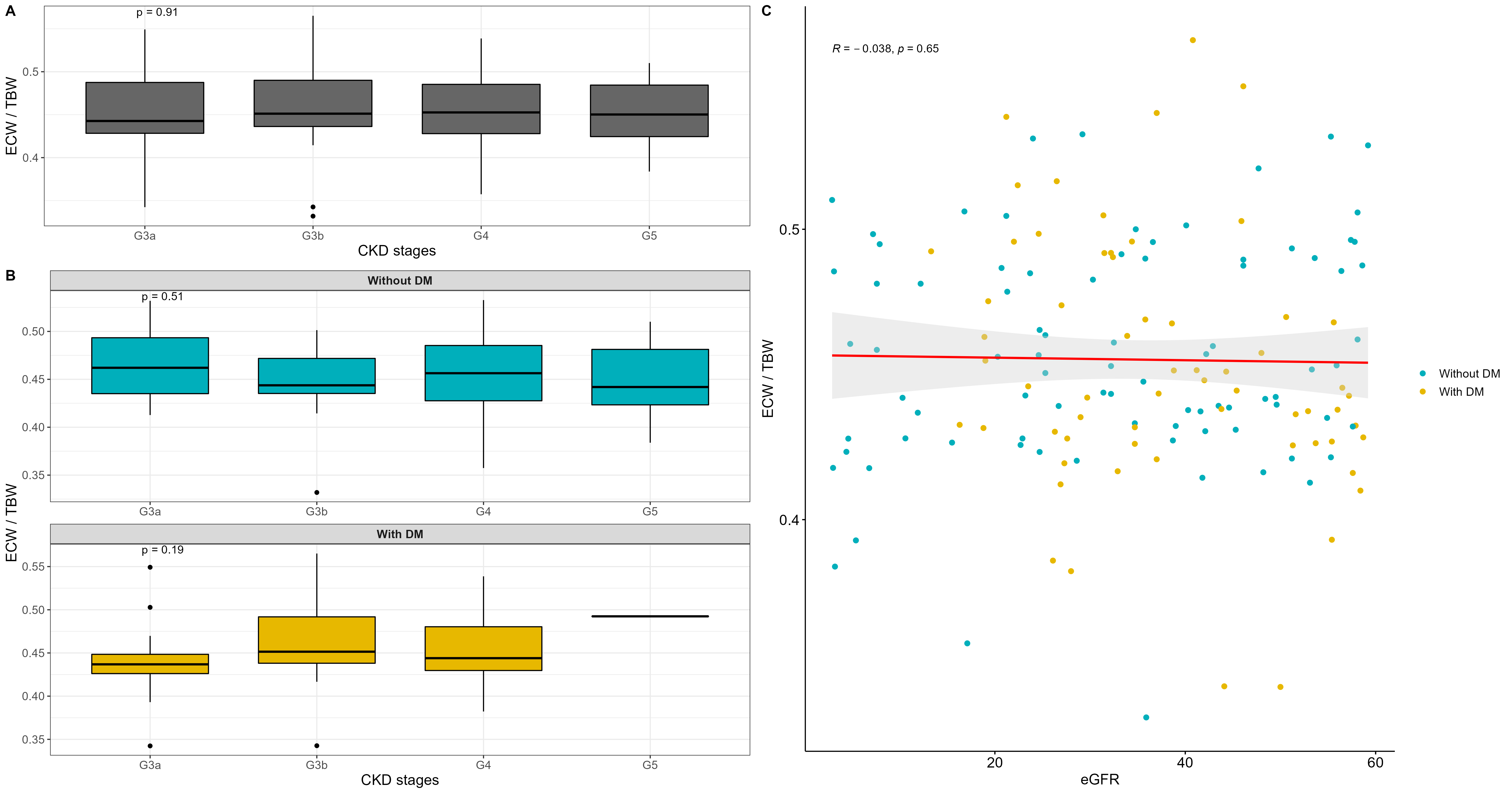

Supplement: Supplementary file 1 [file nutrients-15-02045-s001.zip › CKDstats_ECW_TBW.png]

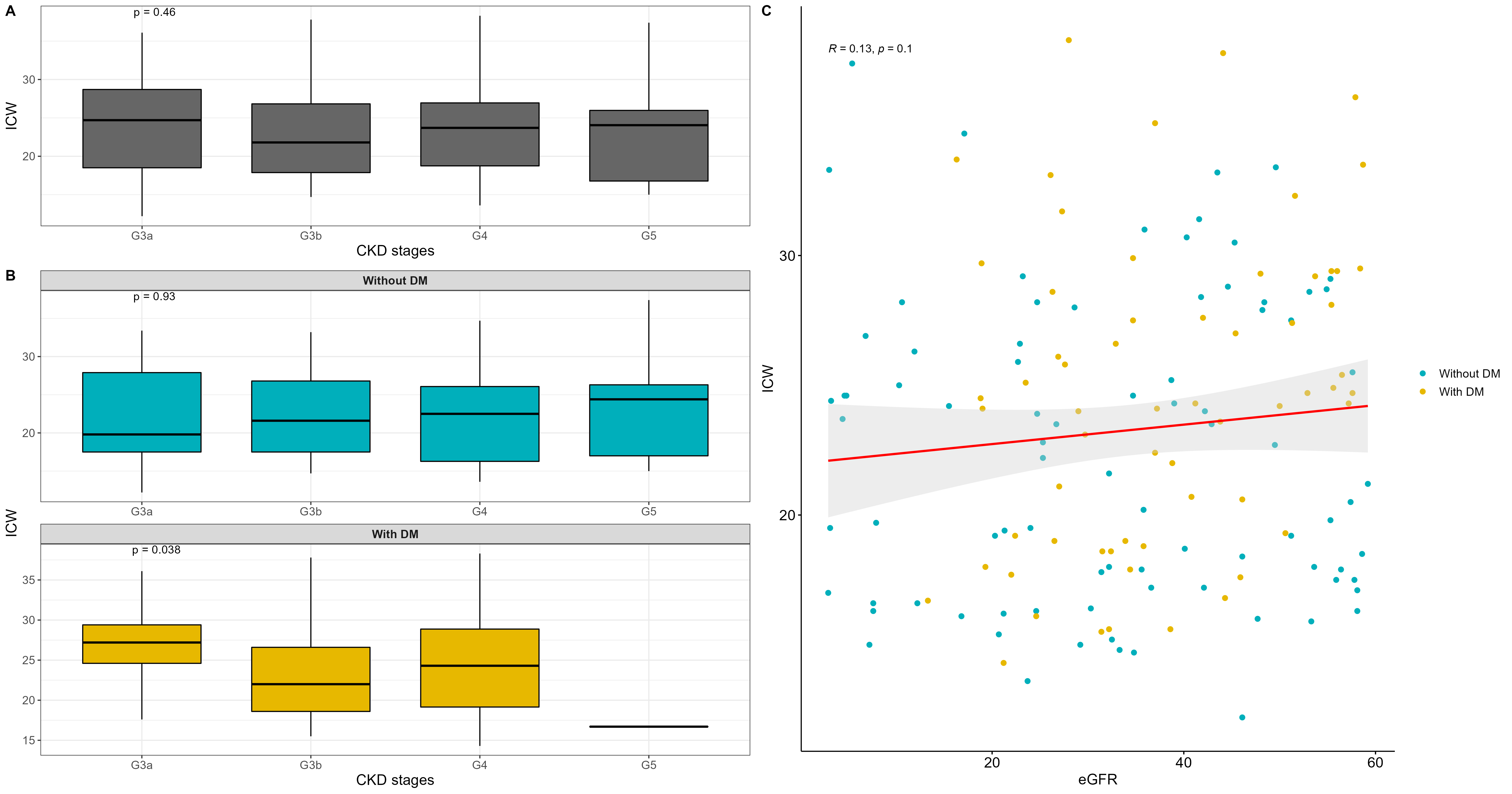

Supplement: Supplementary file 1 [file nutrients-15-02045-s001.zip › CKDstats_ICW.png]

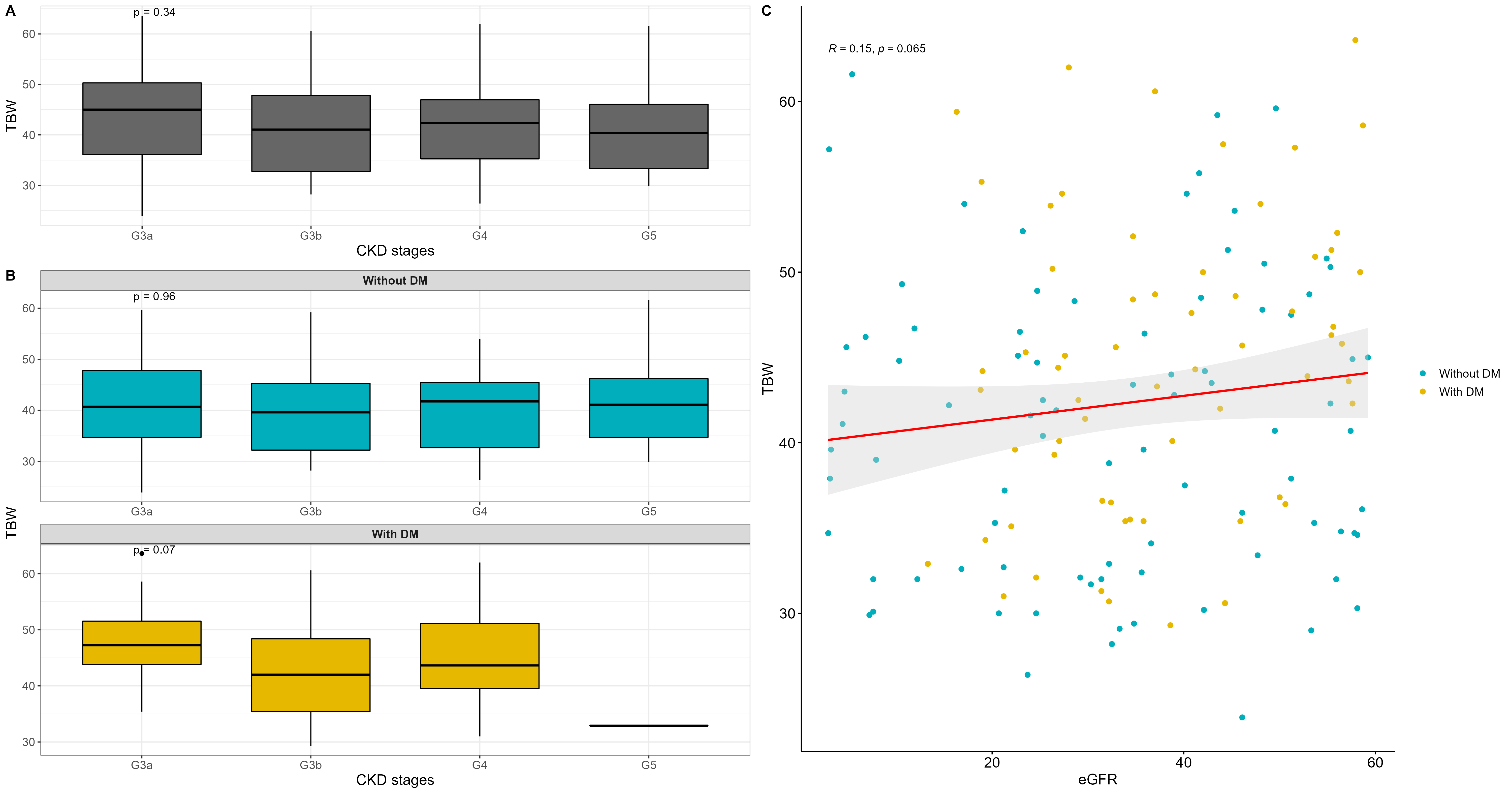

Supplement: Supplementary file 1 [file nutrients-15-02045-s001.zip › CKDstats_TBW.png]

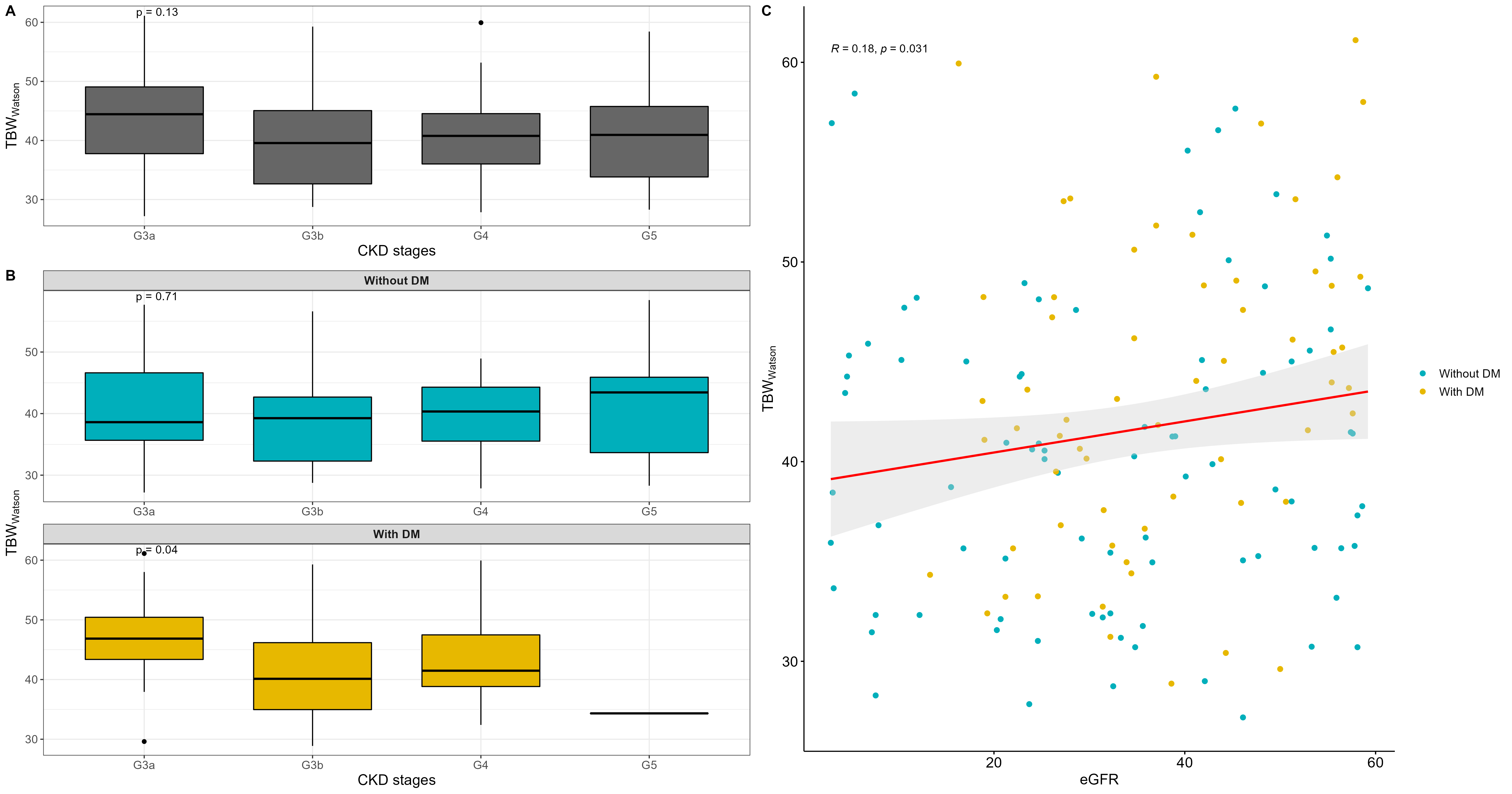

Supplement: Supplementary file 1 [file nutrients-15-02045-s001.zip › CKDstats_TBWwatson.png]

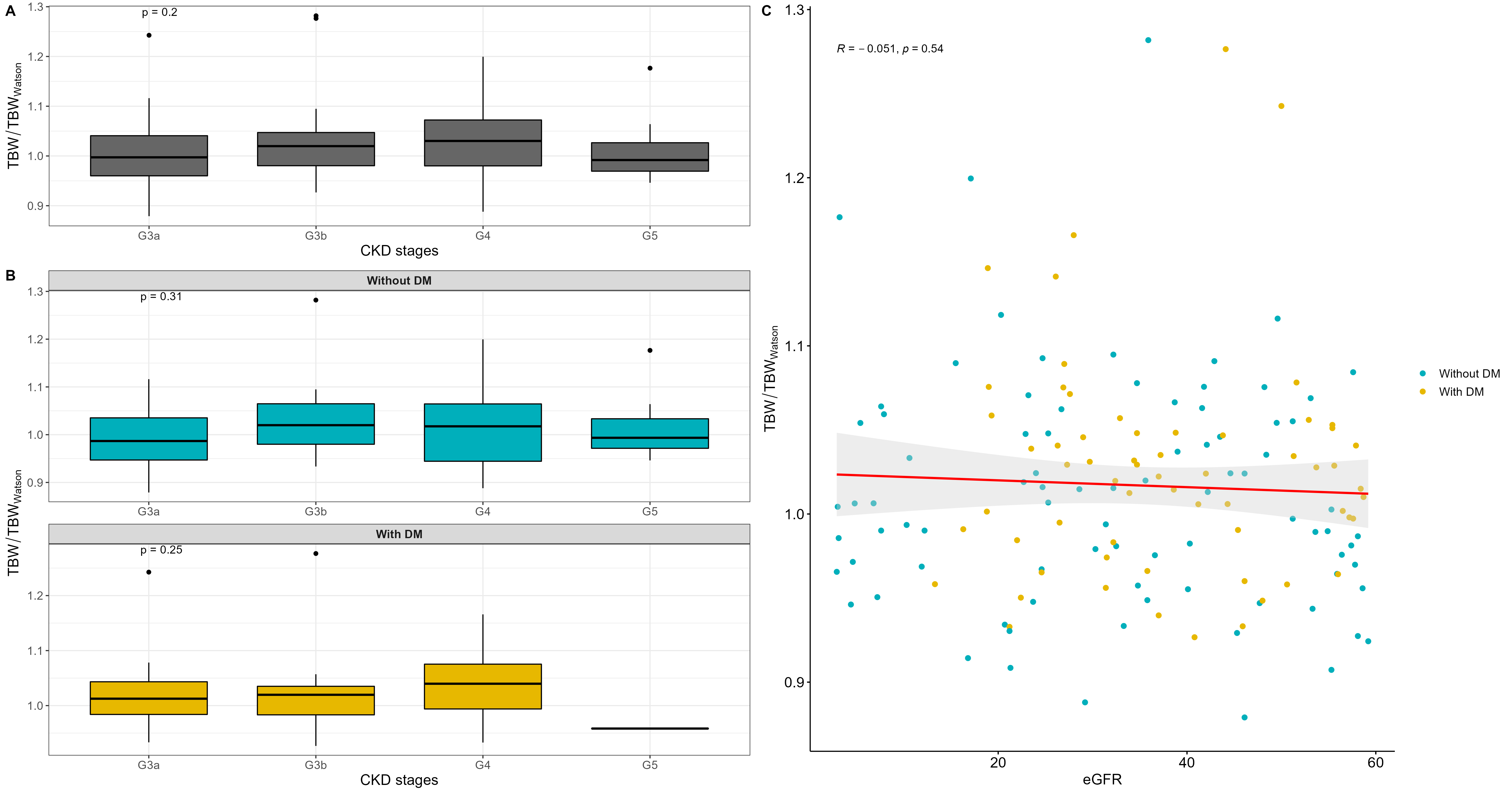

Supplement: Supplementary file 1 [file nutrients-15-02045-s001.zip › CKDstats_TBW_TBWwatson.png]
